# Training in Poor-Law Infirmaries

**Published:** 1907-08-31

**Authors:** 


### Nursing Administration.

### TRAINING IN POOR-LAW INFIRMARIES.

### II. ITS ADMINISTRATIVE DIFFICULTIES.

In a previous article an attempt was made to examine into certain features which are distinctive of the training schools for nurses established in Poor-law infirmaries. It was shown that the points which distinguish infirmary training from that afforded in voluntary hospitals were twofold—practical and administrative. In other words, there are distinctions in the class of work which the nurse is called upon to do, and there are distinctions in the character of the conditions under which the work is performed. The distinctions in the class of work afforded in the infirmary as contrasted with the voluntary hospital are, as we pointed out, more apparent than real, and in so far as they appear a drawback to the making of good nurses, are under able direction susceptible of being turned to good account. The defects which lie on the surface have all their compensating advantages. There is nothing inherent in the conditions of the best Poor-law infirmaries to render their certificated nurses inferior to nurses trained under voluntary management.

We have now to consider in what way the administration, as settled by the Local Government Board, affects the training. Is the peculiar constitution of the Poor-law infirmary calculated to add to the labours of those called to the work of training probationers? Is it favourable to the maintenance of good training schools, or does it render the already arduous work of turning raw material into well-skilled nurses more anxious and intricate?

The matron's position in hospital has often been attacked as one of excessive authority. It is quite true that under certain circumstances the powers entrusted to a matron may be used tyranically. A weak woman finding herself in a position beyond her powers is always prone to degenerate into a despot. But it must be remembered that the matron's position of authority in the hospital household is not a matter which has come down to us from former days, unmodified by modern developments as so many of the official forms of administration have done. It has been evolved in natural course as trained nursing itself has been evolved, by watching the systems which answer best and the causes of failure, and by continual success. In this way it has been proved that to train good nurses, to maintain a high standard of nursing, and secure a spirit of contented loyalty the hospital must have one recognised head over the nursing department. There are numerous counteracting springs which serve to prevent this undivided authority from degenerating into wanton self-assertion. And in practical administration it has been found that the fewer the interruptions supplied to this undoubtedly great responsibility, the better it works for the good of all concerned. The nursing department of a hospital is like a ship. It brooks only one commander. This is unhappily a lesson which the Guardians of Poor-law institutions have never been able to learn. It is inevitable that the Local Government Board should reserve to itself the right of appeal, and it seldom errs in the direction of needless interference. But local government under its least efficient aspect is never so happy as when asserting its fleeting authority, and the tale of the Poor-law infirmaries has been one continuous record of good work accomplished in the teeth of factious opposition from those whose duty it was to recognise and support efficient administrators. Not till the Guardians have learned the lesson which most of them unhesitatingly practise in their private business, of placing one person in authority, and treating the superintendent with generous confidence, will the administration and tone of Poor-law infirmaries as a whole begin to bear comparison with the great voluntary training schools.

There are, however, compensations in the service, even to the limitations of the superintendent's authority. Few matrons depend for their success in dealing with probationers on the grim power in the background of dismissal. True, they may have to bear with girls whose presence is a standing tacit protest against good discipline, and they may be hampered by sisters as coadjutors who have long lost any aspiration with regard to work, except to reduce it to a minimum. But it is under these conditions that the real triumphs of the matron are won, and if compulsion and dismissal are removed from her armoury, it only means to an able administrator that other and more subtle modes of making her will felt will be evolved. The security of tenure, the certainty of a living wage to the end of life provided by the pension system, are restful in their effects, and remove much of that strain which tells in devotion to voluntary hospital work, where unhappily adequate pensions for long service are almost unknown. Moreover, the wear and tear which often falls severely on the matron in raising funds for the voluntary hospital is unknown in the infirmary. There is a kind of interest in perpetually having to raise money, but it is certainly very worrying. All this the Poor-law matron escapes. She escapes, too, all the varied claims on her time which are entailed by strong public interest in the affairs of her institution. It depends upon her temperament whether she regards this as a boon or otherwise. To our mind the infirmary suffers in every possible way from the strange lack of interest in its well-being exhibited by the average ratepayer. A small number of those responsible, a number among whom women Guardians are conspicuous, do sometimes take a real and intelligent interest in the needs of the infirmary. But the majority of these representatives of the ratepayers regard the care of the sick poor merely as a gigantic burden, to be suspiciously watched lest the fads of the philanthropist should make too severe a drain upon the pockets of those who are themselves unable to pay for the benefits of skilled nursing. This strangely indifferent attitude towards the work among the public at large is at the root of many defects in the Poor-law Training Schools, as contrasted with those of the hospitals.

